# Reinventing quantitative genetics for plant breeding: something old, something new, something borrowed, something BLUE

**DOI:** 10.1038/s41437-020-0312-1

**Published:** 2020-04-15

**Authors:** Rex Bernardo

**Affiliations:** Department of Agronomy and Plant Genetics, University of Minnesota, 411 Borlaug Hall, 1991 Buford Circle, Saint Paul, MN 55108 USA

**Keywords:** Plant sciences, Genetics

## Abstract

The goals of quantitative genetics differ according to its field of application. In plant breeding, the main focus of quantitative genetics is on identifying candidates with the best genotypic value for a target population of environments. Keeping quantitative genetics current requires keeping old concepts that remain useful, letting go of what has become archaic, and introducing new concepts and methods that support contemporary breeding. The core concept of continuous variation being due to multiple Mendelian loci remains unchanged. Because the entirety of germplasm available in a breeding program is not in Hardy–Weinberg equilibrium, classical concepts that assume random mating, such as the average effect of an allele and additive variance, need to be retired in plant breeding. Doing so is feasible because with molecular markers, mixed-model approaches that require minimal genetic assumptions can be used for best linear unbiased estimation (BLUE) and prediction. Plant breeding would benefit from borrowing approaches found useful in other disciplines. Examples include reliability as a new measure of the influence of genetic versus nongenetic effects, and operations research and simulation approaches for designing breeding programs. The genetic entities in such simulations should not be generic but should be represented by the pedigrees, marker data, and phenotypic data for the actual germplasm in a breeding program. Over the years, quantitative genetics in plant breeding has become increasingly empirical and computational and less grounded in theory. This trend will continue as the amount and types of data available in a breeding program increase.

## Introduction

The marriage between quantitative genetics and plant breeding, albeit nonexclusive, has reaped benefits for both during the last 100 years. Estimates of genetic variances and heritability in many plant species have increased our understanding of the inheritance and variation of important traits related to yield, quality, and adaptation (Gardner 1963; Matzinger 1963; Hallauer and Miranda 1988). Quantitative genetics principles have led to the design and refinement of breeding methods for continuous traits (Dudley and Moll 1969). Mapping studies have identified major quantitative trait loci (QTL) that have been found useful in crop improvement, and that have helped elucidate the nature of quantitative variation (Kearsey and Farquhar 1998; Bernardo 2020).

That being said, developments in both plant breeding and quantitative genetics in the last several decades should cause us to pause and ponder how quantitative genetics can best be applied in contemporary plant breeding. The purpose of this *Opinion* article is to provide a framework for reflection, discussion, and constructive debate of reasons and ways to reinvent quantitative genetics within the context of current plant breeding. The ideas proposed herein apply mainly to this context and they might not apply to other fields, such as human genetics and animal improvement, where quantitative genetics has also played an important role.

## Origins and foundations of quantitative genetics

Plant breeders may sometimes think that quantitative genetics was developed for the purpose of enhancing plant and animal improvement. After all, most economically important traits in crops and livestock species are quantitative rather than qualitative. However, quantitative genetics was developed not to provide a basis for artificial selection in different species, but to model how variation for continuous traits arises from unknown genes. Quantitative genetics originated more than a century ago in the aftermath of a heated dispute between two groups of eminent English scientists: the biometricians led by Karl Pearson and W.F.R. Weldon, and the Mendelists led by William Bateson (Provine 1971).

The field of biometry began with Francis Galton’s work on how human characteristics, particularly height, were passed from parent to offspring (Galton 1869, 1889). Galton observed a regression toward mediocrity (now known as regression toward the mean) in that tall parents tended to have offspring shorter than them, and short parents tended to have offspring taller than them. For height, Galton estimated that the regression of offspring on mid-parent values was 2/3. To illustrate, suppose the mean height of a father and mother exceeded the mean of the population by 9 cm. The height of their offspring would vary but, on average, was expected to exceed the population mean by 2/3 × 9 = 6 cm. Pearson later analyzed Galton’s data as well as subsequent data (Pearson and Lee 1903), and found that the correlation of the height of fathers and mothers with the height of their sons and daughters had a mean of 0.51. Fraternal correlations were found to be slightly higher (mean of 0.53) than parent–offspring correlations. Overall, these studies by Galton and Pearson suggested that continuous variation in humans is at least partially inherited, although the mechanisms for the transmission of such traits remained a mystery.

These findings by the biometricians disagreed with Mendel’s findings, which were rediscovered independently in 1900 by Hugo DeVries, Carl Correns, and Erich von Tschermak. Mendel’s results indicated a particulate nature of inheritance that led to distinct classes, rather than a continuum of observations for a given trait. The absence of distinct phenotypic classes then suggested to the Mendelists that continuous traits are not heritable. DeVries went to the extent of considering the continuous versus discontinuous nature of variation as a criterion for a trait’s transmissibility (Mather 1949).

In 1918, R.A. Fisher wrote a paper entitled “*The correlation between relatives on the supposition of Mendelian inheritance*” that sought to reconcile the conflicting views of the biometricians and Mendelists. In so doing, this seminal article established the foundations of quantitative genetics as we know it today. The “*on the supposition*“ phrase in the title indicated the conjecture that quantitative traits are controlled by genes that behave in a Mendelian fashion, and the crux of Fisher’s supposition was that continuous variation is the result of the cumulative effects of many such genes. Fisher showed that such a model for quantitative variation could lead to the well-established correlations between relatives found by the biometricians. Fisher initially made two key assumptions to make the model tractable: the individuals mated at random, and each biallelic locus behaved independently of others. These two simplifying assumptions enabled genotype frequencies to be expressed in terms of allele frequencies and vice versa according to Hardy–Weinberg equilibrium, and allowed the cumulative effects across loci to be described in terms of the effect at an individual locus. At several junctures in his article, Fisher investigated the effects of multiple alleles, linkage, assortative mating, and epistasis.

A key feature of Fisher’s derivations was the partitioning of effects into a transmissible and a non-transmissible portion. The transmissible or “additive” portion was due to the effects of individual alleles passed on by a parent to its offspring. These transmissible effects of alleles have since become known as the average effect of an allele (Fisher 1941). The variance due to the average effects of the two alleles at a locus in a diploid has since become known as additive variance (*V*_*A*_). On the other hand, the non-transmissible portion of effects included dominance deviations as well as any environmental effects; to Fisher, dominance deviations were “*a residue which acts in much the same way as an arbitrary error introduced into the measurements*.” The variance due to dominance deviations has since become known as dominance variance (*V*_*D*_). Fisher recognized the existence of effects due to interactions of alleles at different loci and treated such effects as higher-order extensions in a linear model: “*We may use the term Epistacy to describe such deviation, which although potentially more complicated, has similar statistical effects to dominance*.”

Fisher revisited Galton’s concept of parent–offspring regression and derived this regression as being equal to ½*V*_*A*_/(*V*_*A*_ + *V*_*D*_). Because Fisher treated *V*_*D*_ as a residual value that was not any different from random error, we can modify this parent–offspring regression as being equal to ½*V*_*A*_/*V*_*P*_ = ½*h*^2^, where *V*_*P*_ is the phenotypic variance and *h*^2^ = *V*_*A*_*/V*_*P*_ is the narrow-sense heritability. Returning to our height example, suppose the difference between the height of a father and the population mean is denoted by *S*. If the height of the father and mother is uncorrelated (i.e., mating is at random with respect to height), the expected deviation (from the population mean) of the height of the offspring is then equal to ½*h*^2^*S*.

We see this expression as clearly being related to the so-called breeder’s equation of *R* = *h*^2^*S*, where *R* is the response to selection and *S* is the selection differential (Lush 1937). The factor of ½ is due to regression on only one of the two parents, and this situation is equivalent to mass selection without pollen control in plants, for which the genetic gain quantified via *R* is equal to ½*h*^2^*S*. This brief historical summary shows that although quantitative genetics was initially developed as a basic rather than an applied science, quantitative genetics does provide a framework for designing breeding programs that can maximize genetic gain.

## Why reinvent quantitative genetics for plant breeding?

Through the years, scientists have investigated the confluence of the science of quantitative genetics and the process of plant breeding. The 1940s saw investigations of the genetic basis of heterosis (Comstock and Robinson 1948; Robinson et al. 1949) and the development of recurrent selection procedures (Jenkins 1940; Hull 1945; Comstock et al. 1949) to address perceived limitations in line and hybrid development (Jenkins 1934). The 1950s and 1960s have been described as the golden era for quantitative genetics in plant breeding (Gardner 1977), given the research during these two decades on covariances between relatives (Kempthorne 1954; Cockerham 1956); mating designs to estimate *V*_*A*_, *V*_*D*_, and epistatic variance (Comstock and Robinson 1952; Cockerham 1963); empirical estimates of genetic variances in different plant species (reviewed by Gardner 1963; Matzinger 1963; Hallauer and Miranda 1988); genotype × environment interaction (Sprague and Federer 1951); stability analysis (Finlay and Wilkinson 1963; Eberhart and Russell 1966); and index selection for multiple traits (Brim et al. 1959; Pešek and Baker 1969). The 1970s saw the start of investigations of isozymes as markers for quantitative traits (Stuber and Moll 1972; Hamrick and Allard 1975). Long-overdue work started in the 1980s on formal methods to select parents in breeding programs (Dudley 1984). “Bandwagons” related to plant breeding from the 1990s to the present (Bernardo 2016) have included linkage mapping of QTL, association mapping, genomewide prediction (or genomic selection), phenomics, envirotyping, and gene editing.

Plant breeding in the 2020s is markedly different from plant breeding in decades past, and some of the older quantitative genetics approaches described in the preceding paragraph may have become irrelevant. Described below are three reasons for reinventing quantitative genetics as it applies to plant breeding: different expectations, unmet assumptions, and new tools.

### What breeders expect from quantitative genetics has changed

A half-century ago, plant breeders expected quantitative genetics to provide answers to multiple questions (Dudley and Moll 1969) that can be distilled into three:

1. Which germplasm is the most promising?
2. What type of variety or cultivar should be developed?
3. What breeding method should be used?

Methods in quantitative genetics could indeed provide answers to each of the above questions. A set of germplasm that has a superior mean and a large *V*_*A*_ would constitute promising breeding germplasm, and information on the mean and genetic variance can be combined into a usefulness criterion (Schnell 1983) that estimates the mean of a selected proportion of the best individuals in a given population. A large amount of heterosis and *V*_*D*_, as has been found for maize (*Zea mays* L.) yield (Hallauer and Miranda 1988), would indicate that hybrid or synthetic cultivars are suitable. Versions of the breeder’s equation for different types of recurrent selection procedures could be used, along with estimates of *V*_*A*_, *V*_*D*_, and nongenetic variance, to identify which breeding procedures would lead to the largest predicted gain.

The reality, however, is that sufficient answers to the first two questions are obtained without any detailed quantitative-genetic analysis whatsoever. For example, a large set of germplasm can be phenotyped to identify candidates with the best mean performance. Information on germplasm origins or, since the 1990s, data on molecular markers can be used to assess germplasm diversity. Hybrid cultivars are feasible if a hybrid outperforms its parents by a substantial amount, and if hybrid seeds can be produced in a cost-effective manner, with estimates of *V*_D_ being unnecessary. As for the third question, the predicted *R* differs across recurrent selection methods such as recurrent mass selection, half-sib recurrent selection, full-sib recurrent selection, or reciprocal recurrent selection. However, breeders of major crop species such as maize, wheat (*Triticum aestivum* L.), rice (*Oryza sativa* L.), soybean (*Glycine max* (L.) Merrill), and tomato (*Solanum lycopersicum*) have preferred the expediency of a nonrecurrent line and hybrid development system with biparental crosses instead of longer-term recurrent selection with broadbase populations. Because the variance among recombinant inbreds in a biparental cross is constant at 2 *V*_*A*_, there is little or no difference in the gains from line development via different methods (pedigree breeding, bulk method, single-seed descent, or doubled haploids) as long as reliable phenotyping of lines is done at some point during the line development process.

What, then, do current plant breeders expect from quantitative genetics? Today’s plant breeders expect one primary outcome from quantitative genetics: to help identify which candidates have the best genotypic value for a given set of continuous traits, with genotypic value being defined as the expectation of the performance of the candidate in a target population of environments. The candidates would be individual plants, partially inbred lines, or recombinant inbreds in a self-pollinated species such as wheat; testcrosses or hybrids in a cross-pollinated species such as maize; or individual clones in an asexually propagated species such as cassava (*Manihot esculenta*). This singular emphasis on finding lines, hybrids, or clones with the best genotypic value is a much more focused objective than the above three questions from 50 years ago (Dudley and Moll 1969). This singular emphasis suggests that some of the classical methods used in quantitative genetics to address broader sets of questions have outlived their usefulness.

### Classical assumptions in quantitative genetics are unmet in plant breeding

The two main assumptions invoked by Fisher (1918)—random mating and independence of segregating loci—are typically unmet in plant breeding programs. The assumption of random mating is met in species for which recurrent selection is the main breeding procedure, because each cycle of recurrent selection is created by random mating a group of selected individuals in the previous cycle. Recurrent selection is common in forage species such as alfalfa (*Medicago sativa* L.) and perennial ryegrass (*Lolium perenne* L.) but, as mentioned above, not in row crops such as maize and wheat. The F_2_ of a biparental cross between homozygous maize or wheat parents has the same 1:2:1 genotype ratio attained via random mating. Each F_2_ population can therefore be treated as a random-mating population, and *V*_*A*_, *V*_*D*_, and *h*^2^ can be estimated, but such estimates apply only within the given F_2_ population.

In most situations, the entirety of germplasm within a breeding program does not comprise a random-mating population. Maize-breeding germplasm in the United States, for example, includes lines derived from key progenitors, such as A632, B37, B73, Iodent, LH82, Maiz Amargo, Minnesota 13, Mo17, and Oh43 (Troyer 1999; Mikel and Dudley 2006). Maize breeding has focused on developing newer versions of inbreds that maintain these lineages or that combine only a few of these lineages at a time. The preponderance of these key lineages indicates that the maize germplasm in the breeding program cannot, as a whole, be assumed as representative of a random-mating population.

The assumption of linkage equilibrium or independence among segregating loci is particularly problematic (Cockerham 1963). Multiple generations of random mating are needed to achieve linkage equilibrium, especially for closely linked loci. While geneticists are sometimes able to create mapping populations that have undergone multiple generations of random mating (e.g., intermated B73 × Mo17 maize population (Lee et al. 2002)), breeders do not have that luxury in cultivar development programs. The failure in plant breeding to meet the two main assumptions that underlie classical quantitative genetics theory indicates a need to revisit such theory or find ways to circumvent the assumptions.

### New tools and computing capabilities have emerged

Two technological developments have enabled new approaches in both quantitative genetics and plant breeding. First, cheap and abundant molecular markers in different plant species have allowed new types of analysis that were not possible prior to the 1980s. Breeders and geneticists were limited to the use of only up to a few dozen isozyme markers in the 1970s, but the number of available markers increased with the development of restriction fragment length polymorphism (RFLP) markers in the 1980s (Beckmann and Soller 1983). Other types of markers developed after RFLP markers included random amplified polymorphic DNA markers, amplified fragment length polymorphism markers, and simple sequence repeat markers. The number of markers increased drastically with the development of single nucleotide polymorphism (SNP) markers which, unlike previous marker systems, are amenable to high-throughput genotyping platforms (Syvänen 2005). The costs of SNP genotyping have decreased since the 2010s to the extent that in major crop species, the per-sample cost of genotyping (Ertiro et al. 2015) is less than the per-individual cost of multi-environment phenotyping (http://techservicespro.com/).

Second, developments in computers have enhanced data analysis and simulation. G.F. Sprague, a renowned maize breeder who developed the concepts of general and specific combining ability in the 1940s (Sprague and Tatum 1942), once told me that in his day, having a winter nursery was infeasible because yield data from the fall harvest could not be analyzed in time to select candidates to include in a winter nursery. When I started my career with a seed company in 1988, data from a given year’s yield trials were used only for the purpose of selecting the best lines and hybrids that year. Such data were not yet viewed as a valuable contribution to a cumulative, historical data set useful for predicting the performance of candidates in future years. This mindset within the company started to change after I developed genomic best linear unbiased prediction (GBLUP) for single-cross performance in 1994, with the first GBLUP calculations being implemented on a Pentium 90 machine that had 64 megabytes of random access memory (Bernardo 1994). Today’s computers allow large-scale data analysis, such as solving a system of equations with up to 2 million unknowns (Gray 2016), and statistical resampling procedures, such as bootstrapping and cross-validation (Efron 1980), that were not possible before.

## Something old, something new, something borrowed, something BLUE

Reinventing quantitative genetics for plant breeding requires revisiting classical concepts and approaches in quantitative genetics, keeping what remains useful, letting go of what has become obsolete, and considering new ideas. One may question whether a reinvention is needed, because an alternative approach is to simply let the language of quantitative genetics evolve with both use and misuse, to the extent that some current practices might bear little pertinence to the original concepts. We as practitioners would still be able to effectively communicate with each other because we all uniformly follow the same practices. A premise in this *Opinion* article, however, is that if we wish our science to be precise and rigorous, we then should be precise in defining key concepts and rigorous in adhering to them. The stance herein is that it is better to invent new concepts to accommodate new developments rather than to force new developments into classical concepts that might not be able to accommodate them.

Described below are eight main ideas that could underlie neoquantitative genetics for plant breeding. These ideas are organized according to the old English rhyme of “something old, something new, something borrowed, something blue.”

### Old: multiple loci plus environmental effects

As postulated by Fisher (1918), continuous variation will continue to be modeled as being due to the joint effects of multiple Mendelian loci. No assumptions need to be made regarding the number of underlying QTL. For most traits, however, the number of QTL is expected to be large and their individual effects are expected to be small.

The core model for quantitative variation will remain *P* = *G* + *E*, where *P* is the phenotypic value, *G* is the genotypic value, and *E* is a residual value due to nongenetic effects. More specifically, the phenotypic value of genotype *i* in environment *j* is modeled as

1 $${\it{Y}}_{{{ij}}} = \mu + {\it{g}}_{{i}} + {\it{e}}_{{j}} + ({\it{ge}})_{{{ij}}} + \varepsilon _{{{ij}}}$$

where *µ* is the grand mean; *g*_*i*_ is the effect of genotype *i*; *e*_*j*_ is the effect of environment *j*; (*ge*)_*ij*_ is the genotype × environment interaction effect associated with genotype *i* and environment *j*; and ɛ_*ij*_ is the within-environment error. This classical linear model remains unchanged. However, there may be multiple ways to model the *g*_*i*_, *e*_*j*_, and (*ge*)_*ij*_ components. For example, (*ge*)_*ij*_ can itself be modeled as a multiplicative effect obtained as the product of an interaction score due to genotype *i* and an interaction score due to environment *j* (Gollob 1968; Gauch 1988). Envirotyping can be used to model the *e*_*j*_ component, at least in part, as a function of environmental variables such as precipitation and temperature (Cooper et al. 2014).

### Old: identify candidates with the best genotypic value

Plant breeders have always been interested in identifying and selecting candidates with the best genotypic value, and these efforts will intensify as the number of candidates increases due to expansion of breeding programs. Plant breeding has always been predictive in the sense that in the *P* = *G* + *E* equation, the *G* component is predicted from the observed *P*. Predictions of *G* from *P* will obviously become more precise as *E* approaches zero. Methods to make phenotyping more accurate and precise, so that *E* approaches zero, will continue to be important.

Phenotypic data routinely generated in a breeding program and SNP markers have been found useful for predicting the genotypic values of other candidates (see Bernardo (2020) for a review). Such predictions are commonly made via GBLUP, in which SNP markers are used to estimate relatedness among individuals (Lynch 1988; VanRaden 2008), or via an approach such as ridge regression–best linear unbiased prediction (RR–BLUP), in which the effects of each SNP marker are calculated from a set of related individuals (Meuwissen et al. 2001). The GBLUP and RR–BLUP approaches are equivalent when the number of QTL is large, no major QTL are present, and the QTL are evenly distributed across the genome (Fernando 1998; Habier et al. 2007). Given that GBLUP was developed more than a quarter-century ago (Bernardo 1994) and genomewide prediction via RR–BLUP or Bayesian models was proposed nearly 20 years ago (Meuwissen et al. 2001), we must consider both approaches for predicting genotypic value as old.

### Old: continue finding major QTL

Major QTL alleles, such as *Fhb1* for Fusarium (*F. graminearum*) head blight resistance in wheat (Anderson et al. 2007) and *Sub1* for flooding tolerance in rice (Septiningsih et al. 2009), will continue to be useful in cultivar development. A major QTL has an effect large and consistent enough to be meaningful in a breeding program, which implies that a QTL might be considered as major in one breeding program but not in another (Bernardo 2014a). Major QTL may be present for traits such as morphology, phenology, and tolerance to biotic and abiotic stresses, but are likely absent for a highly selected trait such as yield in elite germplasm.

The expected change in the mean value for the trait should be used as the criterion to assess whether or not a marker-trait association represents a major QTL. The *R*^2^ value should not be used as the criterion because a high *R*^2^ value may correspond to too small a predicted change. For example, studies at the University of Minnesota identified a marker with *R*^2^ = 27% for oil concentration in maize (Garcia 2008). The positive QTL allele was predicted to increase kernel oil concentration from 3.5 to 5.5%. Because high-oil maize hybrids with up to 8.0% oil have been commercially available (Lambert et al. 1998), the effect of the QTL is deemed too small for it to be considered as a major QTL in maize breeding (Bernardo 2020).

### New: no need for a reference population in Hardy–Weinberg equilibrium

As mentioned earlier, Fisher’s (1918) assumption of having a random-mating population is typically violated in plant breeding. It is high time to openly acknowledge this fact rather than to pretend, by estimating parameters such as *V*_*A*_ and *h*^2^ in a nonrandom-mated population (Sughroue and Hallauer 1997) or diversity panel, that the assumption of a random-mating population is met. Fisher’s assumption of random mating was a matter of necessity in 1918. In contrast, today’s availability of SNP markers allows breeders to track the transmission of chromosomal segments, and random mating therefore does not need to be assumed. Furthermore, because breeders are mainly interested in identifying candidates with superior genotypic values, there is no need to have a reference population to which inferences would apply—regardless of whether or not such a reference population in Hardy–Weinberg exists.

To illustrate, suppose recombinant inbreds are developed by selfing from a (Parent 1 × Parent 2)F_2_ and from a ((Parent 1 × Parent 2) × Parent 1)BC_1_. Whereas the F_2_ population can be assumed to behave as a random-mating population because the expected genotype ratio of 1:2:1 at segregating loci is the same as that with random mating, the same assumption cannot be made for the BC_1_ population because the expected genotype ratio is 1:1 at segregating loci, with one of the homozygotes not being recovered. At this point, the breeder is simply interested in identifying the best recombinant inbreds, regardless of whether an inbred was derived from the F_2_ or the BC_1_. The breeder has no need to make inferences regarding the mean or *V*_*A*_ or *h*^2^ in the F_2_ or BC_1_, and it is therefore moot that one population is in Hardy–Weinberg equilibrium and the other is not.

The preceding example also brings to light the historical divergence between two schools of thought in quantitative genetics: the Edinburgh/Alan Robertson/Falconer (1960) school that emphasized arbitrary allele frequencies in random-mating, outbred populations versus the Birmingham/Mather (1949) school that focused on crosses between homozygous lines for which allele frequencies are expected to be ½ at segregating loci. While the Edinburgh school has become more prevalent in plant and animal breeding because of its emphasis on artificial selection with arbitrary allele frequencies, the Birmingham school has features that lend themselves naturally to plant breeding as practiced today. At the same time, allele frequencies of ½ are easily accommodated in the Edinburgh framework, and the F_2_ genotype frequencies are those expected with random mating. Both the Edinburgh and Birmingham schools therefore apply to F_2_ populations encountered in plant breeding.

Not needing a reference population also circumvents the issue of whether the candidates are random members of a base population or are a fixed set of lines, clones, or hybrids that are not random members of any population. Suppose the candidates are a set of wheat cultivars and pre-commercial lines with diverse genetic backgrounds. In this situation, the variance component for lines (if the lines were random) is substituted by ∑*c*_*i*_^2^/(*n* − 1), where *c*_*i*_ is the fixed effect of the *i*th candidate and *n* is the number of candidates.

### New: no need to define different types of genetic variances

Classical quantitative genetics as founded by Fisher (1918) focused on breeding value. However, breeding value is of minimal value to plant breeders for two reasons. First, plant breeders are much more interested in genotypic value than on breeding value. The genotypic value is that of the candidate itself, whereas the breeding value is reflected by the mean of the candidate’s progeny when it is mated with random individuals. To illustrate a key difference between animal and plant breeding, the breeding value of a dairy bull (*Bos taurus*) is of utmost importance because a top bull is prized not for its own milk yield (which is zero), but it is prized for the superior milk yield of its female progeny. In contrast, the genotypic value of a wheat line or a cassava clone is of utmost importance because producers would grow the wheat or cassava cultivar itself, rather than the cultivar’s progeny. The ability to genetically replicate plants but not animals therefore plays a key role in this distinction. The foregoing does not imply that breeders are uninterested in the performance of the progeny of an individual: on the contrary, selection of good parents is a key to success in plant breeding. What the foregoing implies is that individuals used as future parents are first and foremost selected on the basis of their superior genotypic value as individuals.

Second, breeding value is defined only when the mate is chosen at random and, as Falconer (1985) indicated, “*The concept of breeding value is shown to have no useful meaning when mating is not random*.” The assumption of random mating, as discussed earlier, is typically violated in plant breeding.

If we accept that the classical concept of breeding value is not meaningful to plant breeders, neither would *V*_*A*_ be meaningful because *V*_*A*_ is the variance among breeding values. The *V*_*D*_ and epistatic variance (*V*_*I*_) would likewise not be meaningful because these variances are derived from the same framework as *V*_*A*_. Therefore, quantitative genetics reinvented for plant breeding would retire the concepts of *V*_*A*_, *V*_*D*_, and *V*_*I*_. On the other hand, plant breeding students will still need to learn about *V*_*A*_, *V*_*D*_, and *V*_*I*_ so that they can understand classical literature and communicate with quantitative geneticists who work with non-plant species, for which these concepts remain important.

What then should be used as a substitute for *V*_*A*_, *V*_*D*_, and *V*_*I*_? The logical alternative is to simply calculate the variance component due to the candidates. In other words, breeders can calculate *V*_F2_ among F_2_ plants; *V*_F3_ among F_3_ lines; *V*_RI_ among recombinant inbreds; *V*_Clones_ among clones; *V*_HS_ among half-sib families; *V*_FS_ among full-sib families; and *V*_SX_ among single crosses. Single crosses are typically made between parents from two heterotic groups that complement each other, and *V*_SX_ can be partitioned into *V*_GCA1_ for the general combining ability (GCA) of parents from the first heterotic group, *V*_GCA2_ for the GCA of parents from the second heterotic group, and *V*_SCA_ for specific combining ability effects. It will be important to not use the symbol *V*_*G*_ for any of these variance components because *V*_*G*_ is traditionally defined as the sum of *V*_*A*_ + *V*_*D*_ + *V*_*I*_. Avoiding the *V*_*G*_ notation will therefore reduce confusion.

Calculating variance components such as *V*_F2_ or *V*_Clones_ or *V*_SX_ has the advantage of being a direct expression of the variation due to genetic effects among the candidates undergoing selection. As previously mentioned, for example, the variance component for recombinant inbreds is *V*_RI_ = 2 *V*_*A*_. The *V*_RI_ therefore quantifies the amount of genetic variation expressed among the candidates, whereas *V*_*A*_ by itself does not. Fisher’s (1918) assumption of linkage equilibrium is unnecessary for defining and estimating variance components for candidates.

Mating designs such as the factorial, nested, and diallel designs have been developed to estimate *V*_*A*_ and *V*_*D*_ (Cockerham 1963). This proposal renders such mating designs obsolete. Variance components due to candidates, as listed above, can instead be estimated by restricted maximum likelihood methods (Dempster et al. 1977; Harville 1977) within the framework of mixed-model analysis, which is described at the end of this paper.

### Borrowed: focus on reliability and the least significant difference

Retiring the concepts of *V*_*A*_, *V*_*D*_, and *V*_*I*_ also means retiring the concept of *h*^2^. This should be of little practical consequence because, as previously mentioned, *h*^2^ measures the proportion of transmissible genetic effects, whereas breeders are more interested in the performance of the candidates themselves rather the performance of their progeny. On the other hand, plant breeders will continue to be interested in the influence of nature versus nurture on quantitative traits. Broad-sense heritability, which is defined as *H* = (*V*_*A*_ + *V*_*D*_ + *V*_*I*_)/*V*_*P*_, has traditionally provided such measure. Calling *H* as a form of “heritability” is oxymoronic because dominance and nonadditive types of epistatic effects captured in *H* are nonheritable from a parent to its offspring.

The concepts of *h*^2^ and *H* have actually been recognized as being muddled in plants as far back as the 1960s (Hanson 1963). The definition of *h*^2^ is straightforward in animals because an individual animal is both the selection unit and the basis for defining *h*^2^. In contrast, an individual plant is the selection unit in mass selection but not in other breeding procedures that have been used in plants. Suppose selection is conducted among half-sib families developed from a random-mating population of perennial ryegrass. The full amount of *V*_*A*_ is expressed among individual plants in the random-mating population. But when *h*^2^ is estimated by rearranging the breeder’s equation as *h*^2^ = *R*/*S*, the numerator of the resulting *h*^2^ has ¼*V*_*A*_ instead of (1)*V*_*A*_ because only a quarter of the *V*_*A*_ is expressed among half-sib families. Hanson (1963) concluded that the definition of heritability in plants “*becomes lost in a maze of confusion*” and he raised the need (but was reluctant) to consider an alternative to *h*^2^ and *H*.

The concept of “reliability” can be borrowed as an alternative measure of the influence of nature versus nurture on phenotypic measurements (Bernardo 2020). Reliability is defined as the consistency of a test or measurement, and reliability has been widely used to gauge the quality of tests in educational, behavioral, and industrial settings (Cronbach 1951). For example, a university admission test is considered reliable if the same student obtains similar scores when taking different editions or versions of the test (assuming that the student’s ability remains the same across retakes). There are several ways of measuring reliability, one of which is *V*_Subjects_/(*V*_Subjects_ + *V*_*e*_), where *V*_Subjects_ is the variance component due to subjects and *V*_*e*_ is the error variance. In plant breeding, reliability can be defined as the variance component due to the candidates divided by *V*_*P*_.

Reliability, which we denote by *i*^2^, is then strictly tied to the selection unit used. If selection is among recombinant inbreds, reliability is *i*^2^_RI_ = *V*_RI_/*V*_P(RI)_, where *V*_P(RI)_ is the phenotypic variance among recombinant inbreds. If selection is among clones, reliability is *i*^2^_Clones_ = *V*_Clones_/*V*_P(Clones)_. The *V*_P_ should be calculated on an entry-mean basis. Suppose that cassava clones are evaluated in *e* environments with *r* replications in each environment. The *V*_P(Clones)_ is calculated as *V*_Clones_ + (1/*e*)(*V*_Clones × Environments_) + (1/*re*)*V*_ɛ_, where *V*_ɛ_ is the within-environment error variance.

Repeatability has been used as a measure of consistency among repeated measurements (Falconer 1960). While reliability is similar to repeatability, the two are different because repeatability can refer to multiple measurements on the same individuals over space (e.g., measurements on multiple fruits from the same tree) or time (e.g., multiple harvests from the same plants), whereas reliability pertains to multiple measurements (e.g., in different environments) that are independent of each other (Bernardo 2020). In plants, repeatability has been commonly used to measure the consistency among multiple harvests in perennial forage species (Casler et al. 2008; Braz et al. 2015). Repeatability has also been proposed to refer to estimates of heritability when “a nonrandom sample of genotypes is evaluated” (Fehr 1987). Such proposal leads to confusion because it adds a second meaning different from the original concept of repeatability as described by Falconer (1960). The concept of reliability therefore fills a long-time void that neither heritability nor repeatability (Falconer 1960) have filled.

While a high *i*^2^ indicates consistent measurements, information on what constitutes a statistically significant difference would also be helpful for the purposes of selection. For example, breeders might be aware that *h*^2^ is about 0.40 for Fusarium head blight resistance, but they might be surprised to find that this level of *h*^2^ could correspond to a line having 0% infection in one test and 7% in a different test. As a standard practice, it would be helpful to report estimates of both *i*^2^ and the least significant difference, e.g., “*Among recombinant inbreds, estimates of reliability and the least significant difference (in parentheses; P* = 0.10*) were* 0.50 (0.72 Mg ha^–1^*) for yield*, 0.70 (0.85%) *for protein percentage, and* 0.40 (8.5%) *for Fusarium head blight incidence*.” Relaxed significance levels (*P* = 0.10 or 0.20) are recommended, given the relative impacts of a Type I error versus a Type II error in cultivar evaluation (Carmer 1976).

### Borrowed: simulation approach to design a breeding program

Plant breeding for major species is like a factory process in which raw materials (germplasm) are input into a manufacturing system (line, hybrid, or clone development) to have products (cultivars) that are marketed and distributed after rigorous testing and quality control (multi-environment trials) (Bernardo 2020). Individual components of a breeding program can be designed quite easily. For example, the parents crossed to form new breeding populations can be selected according to superior performance for multiple traits and SNP diversity between the parents. Estimates of *V*_Clones × Environments_ and *V*_ɛ_ can be used to determine the number of environments and replications per environment needed to detect a given difference for a quantitative trait as being statistically significant. Selection indices can be constructed to include information on the economic weights of different traits. However, these piecemeal approaches do not consider the entirety of a plant breeding program as a system of interdependent processes. Plant breeders would benefit from the availability of tools to design a plant breeding program as a whole.

Such tools will need to be borrowed from other fields. Operations research involves the use of advanced analytics to make better decisions and can be utilized to design breeding processes such as trait introgression (Cameron et al. 2017). Computer simulation has long been advocated for manufacturing systems (Carrie 1988). Instead of calculating *R* for individual crosses via the breeder’s equation, genetic gains for a breeding program as a whole can be simulated vis-à-vis the costs and time required. Simulation packages such as QU-GENE (Podlich and Cooper 1998), AlphaSim (Faux et al. 2016), and DeltaGen (Jahufer and Luo 2018) have been developed to model quantitative variation and selection, and such software has been used to compare breeding schemes (Wang et al. 2003; Jahufer and Luo 2018). In the future, simulation tools need to be able to incorporate existing germplasm, molecular marker data, and phenotypic data as input variables. This means that in a wheat breeding program, for example, the genetic entities in a simulation process would not be generic individuals, but instead would reflect the pedigrees of the actual wheat lines used in the program, along with their associated SNP and performance data for multiple traits.

The incorporation of simulation tools in plant breeding would require changes in a typical plant breeding curriculum, as well as increased collaborationsf with experts in operations research, data science, and simulation of manufacturing systems. Commercial breeding organizations would be apt to hire graduates with expertise in these areas.

### BLUE: mixed-model analysis

Mixed-model analysis, which involves best linear unbiased estimation (BLUE) of fixed effects and BLUP of random effects (Henderson 1975, 1985), has proven to be an effective framework for analyzing phenotypic and SNP data routinely generated in a breeding program. Breeders of row crops conduct balanced experiments by evaluating the same set of candidates in each of several locations in one or more years. However, the entirety of phenotypic data across candidates, locations, and years are highly unbalanced. Mixed-model analysis provides two key advantages: it handles unbalanced data, and it can incorporate information from relatives through one or more covariance matrices of random genetic effects.

The linear model underlying mixed-model analysis can be Eq. 1 or an extension thereof. For example, the linear model can be expanded to include fixed genetic effects due to major QTL (Bernardo 2014b), subpopulations (Yu et al. 2006), transgenes, or different types of cytoplasm, or fixed environmental effects such as level of nitrogen fertilizer or the prior crop grown in the field. The random genetic effects may correspond to lines, clones, or hybrids as in GBLUP, or to SNP markers as in RR–BLUP. Linkage among markers is reflected in the coefficient matrix in RR–BLUP, thereby circumventing the need to assume linkage equilibrium.

The BLUP approach was first used in animal breeding in 1970 to evaluate about 1200 Holstein dairy bulls in an artificial insemination program (Freeman 1991). The use of BLUP in plants started as a trickle, first via shrinkage of estimates of combining ability toward the mean (Melchinger et al. 1987) followed by utilization of information from relatives to predict single-cross performance (Bernardo 1994). It is encouraging that since the 2000s, mixed-model analysis has become familiar to plant breeding students and commonplace in plant breeding programs.

The use of mixed-model analysis decreases the role of genetics and increases the role of statistics in quantitative genetics. This tendency was already observed in animal breeding more than 30 years ago by the statistical geneticist Oscar Kempthorne, who gave the following characterization of animal breeding theory (Kempthorne 1988):

“With the idea that the genes of an individual are random ones of a population of genes with independence between loci, and the idea the environmental effects can be regarded as realizations of independent Gaussian random variables, we see that we have reduced the whole theory to what we in statistics call mixed linear model theory. The outcome is that what is called the theory of animal breeding is reduced to theory of a mixed linear model with fixed effects and independent Gaussian random effects.

So genetics has disappeared from ideation, except that use is made of coefficients of relationship or kinship or “de parenté” (Malécot 1948) or of parentage (Kempthorne 1957).”

The latter paragraph should be modified today because mixed-model analysis now uses SNP marker data in GBLUP rather than pedigree-based coefficients of coancestry via traditional BLUP. The shrinkage factor (*λ*) used in GBLUP, which is equivalent to (1 − *h*^2^)/*h*^2^ in BLUP, can be estimated by a grid search followed by cross-validation to find the *λ* value that maximizes the predictive ability of the model (de Vlaming and Groenen 2015). An estimate of *h*^2^ is therefore not required and, in this context, identifying the candidates with the best genotypic values becomes purely a statistics problem. Overall, reinvention of quantitative genetics as described in this *Opinion* article involves approaches that are computational and empirical, rather than being based on classical theory and assumptions. This trend will undoubtedly continue as increasing amounts of phenotypic and SNP data and additional types of data (e.g., climate or cultural management practices) become available to inform breeding decisions.
